# Disconnecting to recover: how digital detox tourism shapes attention restoration and psychological well-being through perceived environmental restorativeness and mindfulness

**DOI:** 10.3389/fpsyg.2026.1919406

**Published:** 2026-09-03

**Authors:** Wenting Zhang

**Affiliations:** School of Economics and Finance, Xi'an Jiaotong University, Xi'an, China

**Keywords:** attention restoration theory, digital detox tourism, perceived environmental restorativeness, serial mediation, state mindfulness, subjective well-being

## Abstract

Digital detox tourism has become increasingly popular, yet empirical research on its restorative mechanisms remains limited. Drawing on attention restoration theory and the mindfulness–well-being framework, this study used a two-group quasi-experiment combined with a three-wave longitudinal and dose–response design (*N* = 500). Propensity score weighting was applied to a composite path analysis to adjust for baseline differences arising from self-selection. The study examined how digital detox tourism affects attention restoration and subjective well-being through perceived environmental restorativeness and state mindfulness. Latent variable structural equation modeling showed that detox exposure significantly increased perceived restorativeness, which in turn enhanced subjective attention, life satisfaction, and positive affect through mindfulness; all three serial mediation paths were confirmed by bootstrapping, and the association between disconnection intensity and restorative appraisal held across the sample while it did not hold within the detox condition alone. The study further revealed a mechanistic asymmetry between cognitive and affective well-being, in that life satisfaction was driven both by the direct effect of restorativeness and by its indirect effect through mindfulness, whereas positive affect depended almost entirely on mindfulness. This study carries attention restoration theory into a setting of dual digital–physical disconnection and offers a refined test of the “mainly through mindfulness” proposition within the bounds of a single intervention context.

## 1 Introduction

As mobile internet has become deeply embedded in daily life, constant connectivity has shifted from a convenience into a burden. Digital detox tourism—a form of travel centered on the temporary, structured disconnection from digital devices—is a response to this burden and has grown from a niche practice into a sizable and rapidly expanding market segment. Behind this growth lies the accumulation of digital-age psychological stress among working populations. Technostress and digital fatigue have been widely shown to harm workers’ well-being: the technology intensity of digital workplaces can weaken employee well-being through multiple pathways, including constant connectivity, information overload, and fear of missing out (Marsh et al., 2024), and technostress is associated not only with burnout but also with personality traits and lower subjective well-being (Cuadrado et al., 2024). Such digital fatigue is especially pronounced among urban white-collar workers engaged in knowledge work, creating a genuine demand for proactive, periodic disconnection from digital environments. This demand has itself become a notable social phenomenon, and its prevalence among young people, together with calls for digital disconnection, has been documented in empirical research (Salepaki et al., 2025).

Tourism is one of the main settings in which this demand is expressed. Digital detox tourism is regarded as an emerging product whose selling point is disconnection; from early exploratory studies to accounts of individual experiences, the literature shows that travelers seek restoration through temporary disconnection (Scheppe et al., 2025). In the broader tourism well-being literature, digital-free travel has been proposed as a new pathway to improving tourist well-being and has received preliminary empirical support (Hassan et al., 2022). At the same time, the phenomenon has prompted theoretical reflection and critique: some studies examine the internal tensions of digital detox tourism from a philosophy-of-technology perspective (Gong et al., 2023), while others discuss it within the context of responsible tourism design (Saleh, 2025). The practical stakes of this expansion are what render the evidentiary situation pressing, since detox itineraries are marketed upon an explicit therapeutic promise and are purchased by travelers who accept a real constraint upon their behavior on the strength of that promise, while the mechanism by which such a stay would deliver restoration has not been tested against the ordinary restorative effects of travel, so that the traveler’s decision and the operator’s design alike proceed without the evidence that would discipline either.

Although digital detox tourism has attracted growing attention in both industry and academia, the mechanistic understanding of why and how it works remains limited. Existing research has largely remained at the level of motivational description, phenomenological account, or conceptual discussion. A bibliometric and systematic review of digital detox and digital-free tourism notes that the field still lacks a systematic mapping of the relevant mechanisms and that its cumulative and theoretical integration needs strengthening (Hassan and Saleh, 2024); a theoretical framework from an information systems perspective likewise emphasizes that digital detox research needs a clearer theoretical structure and testable causal pathways (Marx et al., 2025). More specifically, although attention restoration theory is often cited as a framework for explaining the restorative effects of digital detox tourism, its empirical operationalization in this context remains scarce: existing studies tend to invoke the theory conceptually but rarely use rigorous designs to distinguish the effect of digital disconnection from the general restorative effects of leaving everyday environments and contacting nature. As a result, even when improvements in the well-being of digital detox travelers are observed, it is difficult to confirm whether they stem from digital disconnection itself or from the vacation and nature exposure common to all travel. Identifying the psychological mechanisms through which digital disconnection translates into attention restoration and well-being, and establishing how far those mechanisms can be told apart from the ones that travel and contact with nature engage in any case, is the core gap this study aims to fill. This gap is especially evident in existing designs: representative studies mostly use cross-sectional designs with behavioral intention as the main outcome. For example, one study uses partial least squares structural equation modeling to examine how mindfulness, psychological well-being, and escape from technostress influence the intention to participate in digital detox tourism (Önder and Topsakal, 2024). Although such studies lay an important foundation, they cannot capture the temporal order of exposure, mediator, and outcome, nor support strong inferences about causal mechanisms.

To address this gap, this study draws on attention restoration theory and the mindfulness–well-being framework and uses a two-group quasi-experiment combined with three-wave longitudinal tracking and a dose–response design to examine the mechanisms through which digital detox tourism affects attention restoration and subjective well-being via perceived environmental restorativeness and state mindfulness. What sets the present study apart from prior work is the conjunction of a concurrent comparison of ordinary leisure travel with a three-wave ordering of exposure, mediator and outcome and with a continuous representation of disconnection intensity, a conjunction that allows the restorative contribution of a detox stay to be examined apart from the vacation effect that any trip affords and allows the mechanism to be traced rather than inferred. The study makes three expected contributions. Theoretically, it proposes and tests the dual-disconnection hypothesis, which carries the “being away” dimension of attention restoration theory from physical displacement into a setting where digital and physical withdrawal coincide, and it supplies dose–response evidence for that reading within one intervention context rather than a replacement for the existing account. In terms of mechanism, it distinguishes the cognitive and affective components of subjective well-being and examines whether the restorativeness–mindfulness pathway acts differently on the two, thereby offering a refined test of the “mainly through mindfulness” proposition. Methodologically, it sets digital detox travel against a concurrent comparison of ordinary leisure travel rather than against no travel at all, which separates the restorative contribution of a detox stay from the vacation effect that any trip affords, and it represents the exposure continuously so that restorative gain may be examined against the degree of disconnection attained rather than against group membership alone, which addresses the field’s longstanding problem of insufficient empirical operationalization. The comparison holds the fact of travel constant without holding the intensity of contact with nature constant, and the boundary this sets upon the inference is stated in Section 5.4.

## 2 Theoretical background and hypotheses

**Figure 1:**
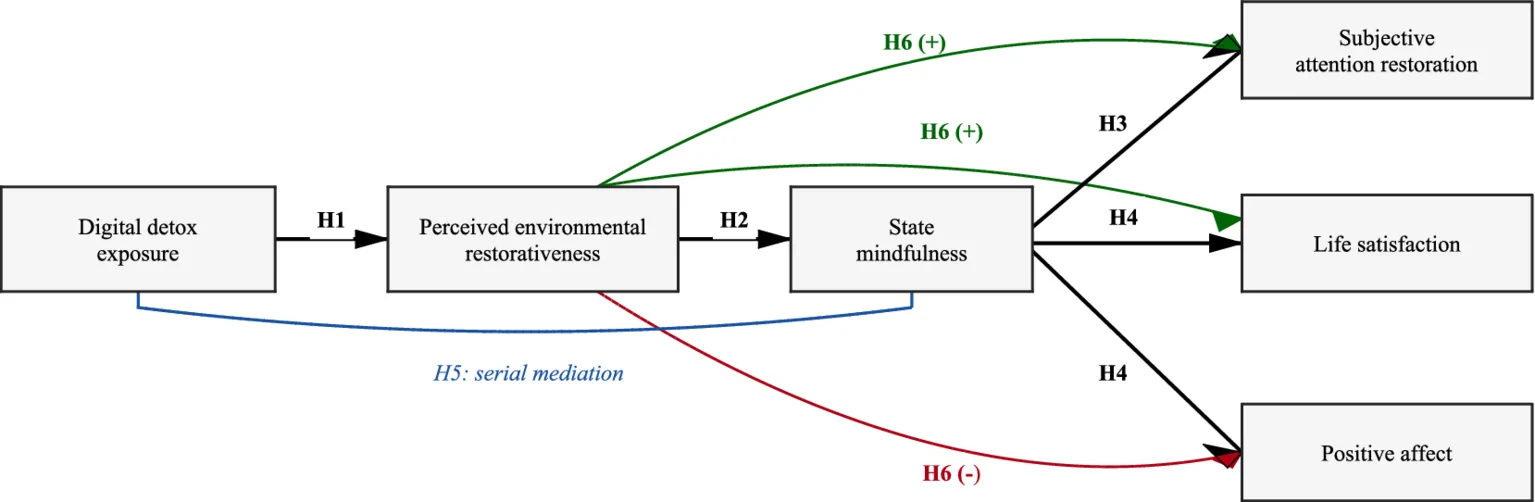
Theoretical conceptual model of the dual-disconnection hypothesis.

The framework of this study is assembled from two theoretical components that address different segments of a single causal chain rather than from a loose aggregation of adjacent literatures. Attention restoration theory specifies the conditions under which an environment relieves the demands placed on directed attention and therefore governs the front end of that chain, namely why the withdrawal of habitual digital and physical demands should raise the extent to which a setting is appraised as restorative. The mindfulness–well-being framework specifies how a present-centered mode of awareness converts an experienced state into evaluative and affective benefit and therefore governs the back end, namely why an environment appraised as restorative should improve how a life is judged and how a moment is felt. The two components meet at a single conceptual seam, since the fascination and compatibility that define a restorative appraisal describe precisely the conditions under which attention can rest on present experience without effortful control, so that perceived environmental restorativeness is the appraisal of the setting while state mindfulness is the psychological state that such an appraisal affords. The digital detox literature is drawn upon here to characterize the exposure rather than to supply an independent explanatory mechanism, and the well-being literature is drawn upon to justify treating the outcome as two components rather than one, an allocation that keeps the whole of the explanatory burden on the restoration–mindfulness sequence. The conceptual model that results, together with the paths it implies, is shown in Figure 1.

### 2.1 Attention restoration and perceived environmental restorativeness

This study takes attention restoration theory as its theoretical starting point. The theory holds that voluntary, directed attention is a depletable resource, and that environments rich in “soft fascination” can restore it; recent integrative work on rest and attention restoration under high cognitive load further consolidates this argument (Schumann et al., 2022). Restorative environments are typically characterized by four interrelated qualities—being away, extent, fascination, and compatibility—and the extent to which individuals perceive these qualities is captured by perceived environmental restorativeness. Existing evidence indicates that these perceptions are themselves structured along the dimensions posited by the theory, and that even in controlled and simulated natural settings, being away and fascination are prominent components (Rhee et al., 2024).

One qualification deserves particular note, concerning the relationship between perceived restoration and performance-based restoration. Experimental studies show that natural scenes are consistently judged as more restorative than urban scenes, yet the corresponding gains in objective attention performance are relatively limited and unstable (Johnson et al., 2022). It is this dissociation that leads this study to model subjective attention restoration and objective attention as separate, independent outcomes, rather than assuming that the two necessarily covary.

In tourism, the restorative attributes of nature-based destinations have been shown to shape recreationists’ experiences and place perceptions, thereby extending the explanatory scope of restoration theory from laboratory and everyday settings to travel settings (Tang et al., 2024). This extension is especially pertinent for high-stress urban populations, among whom perceived environmental restorativeness has been found to be associated with the motivations and expectations of restorative tourism (Ding and Xu, 2024). Building on this, this study treats digital detox tourism as an intensified form of restorative travel and proposes that the removal of digital stimuli enhances individuals’ perception of the environment as restorative.

### 2.2 Mindfulness as a restorative pathway

A restorative environment is unlikely to act directly on well-being; its effect is better theorized as transmitted through the psychological states the environment cultivates. State mindfulness—a present-centered, nonjudgmental mode of awareness—offers a plausible pathway, because the conditions that make an environment restorative also nourish the openness of attention characteristic of mindful experience. Network-analytic evidence linking nature connectedness, mindfulness, and well-being supports the proposition that these constructs form an interconnected system rather than independent influences (Capizzi and Kempton, 2023). Review evidence further indicates that the well-being benefits of combining nature exposure with mindful engagement exceed the sum of either component alone, reinforcing the view that restorativeness and mindfulness operate synergistically (Smith et al., 2024).

The selection of perceived environmental restorativeness and state mindfulness as the core mediators follows from the position each occupies within this sequence rather than from the frequency with which the two appear in adjacent research. Perceived restorativeness is the only construct defined over properties of the setting as the traveler appraises them, which makes it the point at which an exposure defined by what has been removed can register psychologically at all, whereas state mindfulness is the only construct describing the manner in which attention is deployed during the stay, which makes it the point at which an appraised property of a setting becomes an experience capable of carrying benefit beyond it. Alternative mediators do not occupy these positions, since positive affect is modelled here as an outcome and would otherwise restate the affective component of well-being under another name, while reduced rumination, psychological detachment and perceived autonomy are each more readily understood as further consequences of an environment that no longer commands directed attention than as competing origins of that release, so that admitting them would lengthen the chain downstream rather than displace its core, an omission that claims parsimony rather than exclusivity and is returned to in Section 5.5. The proximity of the two mediators warrants an equally direct statement, since an appraisal of a setting and a state of awareness that the setting affords are necessarily associated and a high association is anticipated by the framework rather than embarrassing to it. The two remain distinguishable in kind, restorativeness being predicated of the environment and mindfulness of the person, and the empirical record bears the distinction out, since the heterotrait–monotrait ratio stays below the conventional threshold and, more tellingly, the two carry opposite signs into the positive affect model, where mindfulness contributes strongly and positively while the residual portion of restorativeness contributes negatively (Section 4.4). Constructs that were empirically redundant could not behave in this manner, though the association is close enough that the Fornell–Larcker comparison for this pair is not satisfied, a qualification recorded in Section 4.3 and carried into Section 5.4.

The downstream link from mindfulness to well-being has been directly supported by within-person mediation evidence: during an intervention period, day-to-day increases in state mindfulness are associated with subsequent improvements in mental health and well-being (Verhaeghen et al., 2026). Such within-person findings are valuable because they fit the temporal sequence implied by the present model—restorative experience precedes mindful awareness, which in turn precedes well-being outcomes.

This study treats well-being as a multidimensional construct, comprising both a cognitive component represented by overall life evaluation and an affective component represented by positive affect. The two are conceptually related yet distinct, and this study models them as separate outcomes—a separation also supported by the measurement model reported in Section 4.3. Distinguishing them is substantively meaningful, because the two components differ in the informational basis upon which each is formed and should on that account stand in different relations to the pathway proposed here. A judgement of life as a whole is deliberative and retrospective, drawing upon standards of comparison and upon the remembered coherence of an episode, so that an environment recalled as separate from everyday demands can inform such a judgement without any requirement that mindful awareness have been sustained at the time. Momentary positive affect, by contrast, is constituted within the experience itself and possesses no comparable retrospective basis, so that the pathway is expected to reach it only insofar as the setting was actually attended to in a present-centered manner. The anticipated asymmetry follows from this difference rather than from the observed results, it is stated as Proposition 3 and tested as H6, and the measurement evidence reported in Section 4.3 shows the separation to be sharper than the conceptual argument alone would require.

### 2.3 The dual-disconnection hypothesis

Integrating the threads above, this study proposes the dual-disconnection hypothesis. Its central claim is that, in the digital age, the restorative value of travel comes not only from physical “being away” but also from simultaneous digital disconnection, and that this dual disconnection translates into well-being mainly by enhancing perceived restorativeness and cultivating mindfulness. The three propositions below define the conceptual, testable core of the hypothesis; their corresponding statistically testable forms are given as specific hypotheses in Section 2.4.

*Proposition 1*. In a digitally saturated society, the “being away” dimension of restoration has expanded from mere physical displacement to dual digital–physical disconnection; withdrawal from the habitual digital environment, together with withdrawal from the habitual physical environment, constitutes a distinctive form of psychological distancing. The extension of restoration theory to travel settings provides a basis for this expansion (Tang et al., 2024).

*Proposition 2*. Digital detox travel, set against ordinary leisure travel, produces greater indirect gains in attention restoration and well-being mainly through stronger perceived restorativeness and mindfulness along the mediation chain, and these gains increase with the intensity of digital disconnection. The proposition concerns the detox stay as a composite whose digital and environmental elements are conjoined in practice rather than the digital element taken in isolation, and the extent to which disconnection can be credited apart from the conditions accompanying it is a question that the design of this study bounds rather than settles (Sections 3.1 and 5.4).

*Proposition 3*. The effects of perceived environmental restorativeness on the outcomes are largely transmitted through state mindfulness; for affective outcomes, this mindfulness-mediated indirect path is expected to be dominant, rather than acting on the outcome directly and independently of the mindfulness state it induces.

### 2.4 Hypothesis development

To make the above propositions statistically testable, this study operationalizes them as six hypotheses. The correspondence is as follows: Propositions 1 and 2 are jointly operationalized by H1 (the effect of exposure on perceived restorativeness, with continuous disconnection intensity capturing the “dose” logic); H2 to H5 characterize the restorativeness–mindfulness mediation mechanism presupposed by the propositions and its transmission to each outcome; and the “mindfulness-dominant” claim of Proposition 3 is tested directly by H6 through competing paths.

The premise that digital detox travel strengthens the restorative qualities of an environment follows from the expanded “being away” logic stated in Proposition 1: when habitual connectivity is suspended, the destination is more readily perceived as a domain separated from everyday demands. Because exposure is operationalized both as a group contrast and as continuous disconnection intensity, the relationship is expected to hold under either operationalization (Ding and Xu, 2024).

*H1.* Digital detox exposure increases perceived environmental restorativeness.

Restorative environments are theorized to evoke mindful awareness, because the fascination and compatibility that define restorativeness gently direct attention toward present experience (Rhee et al., 2024).

*H2.* Perceived environmental restorativeness increases state mindfulness.

Insofar as mindful awareness reflects the restoration of directed attentional control, it should accompany the restoration of subjective attention, thereby complementing the account of restored directed attention (Schumann et al., 2022; Johnson et al., 2022).

*H3.* State mindfulness increases subjective attention restoration.

The well-being benefits of restorativeness and mindful experience extend to both evaluative and affective domains, encompassing both satisfaction with life and the experience of positive affect (Capizzi and Kempton, 2023; Smith et al., 2024).

*H4.* State mindfulness increases subjective well-being (comprising life satisfaction and positive affect).

Connecting the paths above forms an indirect process: exposure shapes restorativeness, restorativeness cultivates mindfulness, and mindfulness in turn supports the outcomes; this is consistent with within-person mediation evidence for state mindfulness (Verhaeghen et al., 2026).

*H5.* The effects of digital detox exposure on subjective attention restoration, life satisfaction, and positive affect are serially mediated by perceived environmental restorativeness and state mindfulness.

Proposition 3 implies a testable contrast between the mindfulness-mediated indirect path and the residual path by which restorativeness reaches the outcomes while bypassing mindfulness. This hypothesis is framed competitively, with the mindfulness-mediated effect expected to be at least as large as the direct effect and to dominate for affective outcomes.

*H6.* The indirect effect transmitted through state mindfulness is greater than the direct effect of perceived environmental restorativeness that bypasses mindfulness.

## 3 Methods

### 3.1 Study design and sample

This study used a two-group quasi-experiment combined with three-wave longitudinal tracking and a dose–response design. The treatment group comprised travelers who participated in a digital detox camp of at least 3 days, with average daily screen use restricted to within 1 h and mobile devices held in central storage; the comparison group comprised ordinary leisure travelers during the same period whose itineraries likewise contained natural elements, though not at a comparable intensity. The three measurements followed the temporal sequence implied by the study model: T1, 1 week before departure, collected baseline and predictor variables; T2, on the day the trip ended, collected the mediators (perceived environmental restorativeness and state mindfulness); and T3, 4 weeks after the trip ended, collected the outcomes (subjective attention restoration, life satisfaction, and positive affect). This timing placed the mediator measurements before the outcome measurements, providing a temporal order for mediation inference.

The detox stays were run by three commercial operators of digital detox and nature experience camps, the first situated in mountain forest in the southwest, the second in coastal wetland in the east and the third in hilly grassland in the north, contributing 120, 100 and 80 participants, respectively. Lodging took the form of cabins sleeping four to six, tented quarters or hostel-style rooms against ordinary hotels and guesthouses in the comparison condition, and the price of the two was of a similar order, from about 2,200 to 2,800 yuan per person against about 2,100–2,700, so that cost does not stand as a plausible source of the contrast. Planned duration ran from three to 7 days with a mean of 4.40 days, the two conditions differed appreciably before adjustment with a standardized difference of 0.98, and weighting reduced that difference to 0.04 (Table 1). Daily activity in the detox condition centred upon hiking, nature observation, collaborative tasks, camp labour, handicraft and evening sharing, and in the comparison condition upon visiting scenic sites, photography, unstructured free time, dining and shopping, so that the two differ in more than the digital protocol and the consequences for what the group contrast identifies are stated in Section 5.4. No formal mindfulness instruction, no scheduled period of silence and no meditation session formed part of any camp programme, so that the state mindfulness measured at T2 was not induced by training and stands as a state arising from the setting and from the withdrawal of connectivity rather than as a component of the intervention. Assignment was not random and arose from the traveler’s own booking decision, which is the reason the analysis is weighted rather than treated as experimental.

**Table 1:** Standardized mean differences of representative covariates (before weighting/after IPTW).

Covariate	SMD before weighting	SMD after IPTW	Balanced
Planned trip days	0.98	0.04	Yes
T1 screen time	0.40	0.08	Yes
Burnout	0.27	0.03	Yes
Smartphone addiction	0.23	0.05	Yes
Baseline well-being	0.24	0.07	Yes
Occupation = manufacturing/engineering	0.18	0.02	Yes
City tier = third-tier or below	0.16	0.001	Yes
Age	0.002	0.06	Yes

The three-day floor was an operational criterion rather than a threshold carrying an established theoretical value, since restorative gain behaves as a function of dose rather than as a step change at any particular point (Bell et al., 2025), and the value was set so that a qualifying stay would span at least two full nights while remaining compatible with the length of commercially available detox itineraries. Stays shorter than 3 days are consequently absent from the observed dose range, a point recorded in Section 5.4.

**Figure 2:**
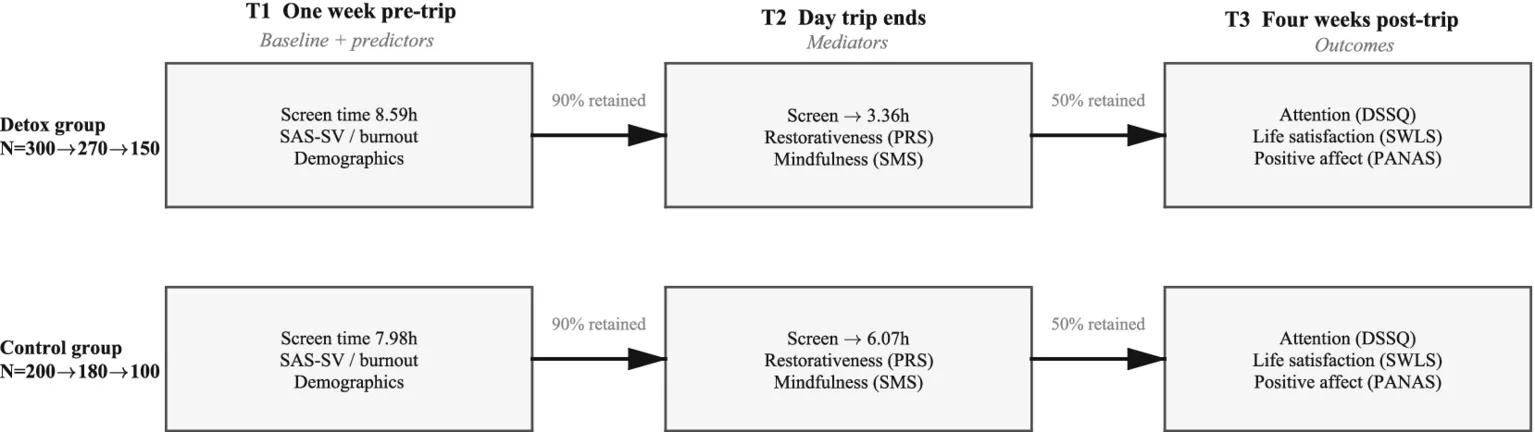
Study design and timeline of the three-wave longitudinal measurements (two-group quasi-experiment).

A total of 500 participants were recruited at baseline, of whom 450 completed T2 and 250 completed T3. Retention rates were identical for the treatment and control groups at both follow-ups (90% at T2 and 50% at T3). Screen use was reported by participants from the usage statistics recorded by the operating system of their own device under its screen time or digital wellbeing function rather than estimated from recollection, which yields a figure independent of retrospective judgement while remaining dependent upon the participant’s transcription of it. Compliance within the detox condition was maintained through a sealed-bag procedure under which each device was numbered and sealed upon arrival and every deposit and retrieval was entered with its time upon a signed register, laptops and tablets were not to be brought and were sealed under the same procedure where a participant had brought one, network-enabled watches were sealed while unconnected timepieces remained with the participant, and retrieval for urgent matters was permitted upon request and was recorded together with its reason, the hour of retrieval and the hour of return. The change in screen use between T1 and T2 documents the implementation of the protocol rather than an independent behavioral response to it, since central storage of devices renders a reduction in use a designed property of the detox condition, and the figure is accordingly reported as a check upon the fidelity of implementation rather than as a manipulation check in the conventional sense. Average daily screen use in the detox condition fell from 8.59 h at T1 to 3.36 h at T2, and the residual use is consistent with the return of devices at the close of the stay, on which day the T2 measurement was taken, rather than with any failure of the protocol. Screen use in the comparison condition fell over the same interval from 7.98 to 6.07 h, which indicates that ordinary leisure travel of itself displaces an appreciable part of habitual digital engagement and that the contrast between the two conditions is therefore one of degree rather than one of kind, and it is on this account that the continuous index of disconnection intensity is reported alongside the binary contrast rather than in place of it, the two bearing upon the same variation and neither identifying the exposure independently of the conditions accompanying it (Sections 3.3 and 4.6). Sample composition together with the fidelity of the screen-time protocol is shown in Table 2. The overall study design, the timing of the three-wave measurements, and the variables measured at each wave are shown in Figure 2.

**Table 2:** Sample retention and fidelity of the screen-time protocol.

Group	T1 (*N*)	T2 (*N*)	T3 (*N*)	Screen time T1	Screen time T2	Screen time T3	T2 retention	T3 retention
Digital detox tourism	300	270	150	8.59	3.36	7.12	0.90	0.50
Ordinary leisure tourism	200	180	100	7.98	6.07	7.56	0.90	0.50
Total	500	450	250	—	—	—	—	—

Screen time is the daily average (hours).

### 3.2 Data sources and covariate balance

Data were obtained through cooperation with digital detox camp operators and through the parallel recruitment of ordinary leisure travelers. Travelers who had enrolled in a qualifying detox stay were approached by a research assistant through the operator’s messaging group, by short message or by electronic mail, and the baseline questionnaire was completed between three and 7 days before departure, whereas comparison participants were recruited over the same period from university communities, from travel agency communities and through an online survey panel, drawn from travelers who had booked leisure trips containing natural elements. Eligibility required an age between 22 and 55, ownership of a smartphone together with habitual daily screen use of no less than 3 h over the preceding week, a booked trip of at least 3 days containing natural elements, the capacity to complete three waves of measurement and written informed consent, while travelers were excluded where severe visual or auditory impairment was present, where crisis intervention or psychiatric admission had been received recently, where the questionnaire could not be understood, where the principal activities of the trip were not in the event attended, where a key variable was missing, or where the quality of response failed the checks described below. Participation was compensated by an electronic voucher of ten yuan for each valid wave completed, amounting to thirty yuan across the three, and enrollment was voluntary throughout, with no traveler’s itinerary contingent upon taking part. Recruitment yielded 300 participants in the detox condition and 200 in the comparison condition (Table 2), whose ages ran from 22 to 55 with a mean of 34.09 and stood almost identical across the conditions (detox M = 34.09, SD = 6.37; comparison M = 34.08, SD = 5.82), and whose baseline daily screen use ran from 3.50 to 12.92 h with a mean of 8.35 (Table 3). This study was reviewed and approved by the Ethics Committee of Xi’an Jiaotong University (approval number: XJTU-2025-216) and followed the relevant principles of the Declaration of Helsinki. All participants signed written informed consent before participation; because the study involved central storage of mobile devices during the trip, the consent form specifically described the manner, duration, and retrieval arrangements for device storage, and participants could withdraw at any time without affecting their trip arrangements. All three waves were administered as an online questionnaire hosted on the Wenjuanxing platform and completed upon the participant’s own device, the T2 questionnaire being answered on the day the stay ended, at which point devices had been released from sealed storage. Each participant was identified across waves by an anonymous code generated at enrollment rather than by name or contact details, responses were held separately from the consent records, and access was confined to the author, with no response available to camp operators or to the recruiting communities at any stage. Response quality was safeguarded by two attention-check items embedded among the scales, by a minimum completion time below which a submission was discarded, by the inspection of address and device identifiers for duplicate submission, and by the identification of invariant response patterns. Of the 548 questionnaires returned at T1, 48 were discarded for failure of an attention check, for completion below the time threshold, for invariant responding, for missingness upon a key variable, or for an identity code that could not be matched across waves, which leaves the 500 baseline participants reported in Table 2. Because this study used a quasi-experimental design in which group assignment was not random, participants’ self-selection could create baseline differences between the two groups. To address this, the study used propensity score methods to adjust for observable demographic and baseline covariates. The core logic of propensity score weighting is to estimate, from individual characteristics, the probability of receiving a given exposure, and to weight the sample by the inverse of this probability, thereby constructing a pseudo-population in which confounders tend to be balanced across groups (Chesnaye et al., 2022). Inverse probability of treatment weighting served as the primary adjustment for self-selection and propensity score matching as a robustness comparison, both applied to the composite path analysis described in Section 3.4 rather than to the latent models reported in Section 4.4.

**Table 3:** Covariate coding and distribution (*N* = 500, no missing values).

Covariate	Type	Values/distribution
Age	Continuous	22–55 years; M = 34.09, SD = 6.15
Sex	Categorical	Female 281; Male 219
Education	Categorical	Bachelor’s 327; Master’s or above 121; Junior college or below 52
Marital status	Categorical	Married/cohabiting 274; Unmarried 214; Other 12
Monthly income	Categorical	8,000–14,999: 216; 15,000–24,999: 141; Below 8,000: 91; 25,000 and above: 52
Occupation	Categorical	Internet/IT 117; Education/research 93; Other white-collar 89; Finance/consulting 82; Manufacturing/engineering 66; Culture-tourism/services 53
City tier	Categorical	First-tier/new first-tier 279; Second-tier 150; Third-tier or below 71
Prior detox experience	Binary	M = 0.18
T1 screen time	Continuous	3.50–12.92; M = 8.35, SD = 1.58
Planned trip days	Continuous	3–7; M = 4.40, SD = 1.19
T1 smartphone addiction (SAS-SV)	Continuous	M = 3.65, SD = 0.71
T1 burnout	Continuous	M = 3.86, SD = 0.64
T1 baseline attention	Continuous	M = 3.07, SD = 0.75
T1 baseline well-being	Continuous	M = 3.03, SD = 0.72
T1 backward digit span	Continuous	2–10; M = 6.60, SD = 1.23

The covariates entered into the propensity score model and their distributions are shown in Table 3. Notably, all covariates had no missing values at T1, which reduced the uncertainty arising from additional imputation due to missing covariates. Because this study involved attrition over longitudinal follow-up, the applicability and limitations of propensity score weighting in handling selection bias due to follow-up attrition—beyond balancing baseline—have been noted in prior methodological discussions, requiring careful specification of the weighting model and cautious interpretation of results (Metten et al., 2022). Extreme weights can inflate variance and distort estimates, so this study applied weight truncation in the robustness step, a practice consistent with the recommendation to improve the robustness of weighted estimates by post-calibrating the propensity scores (Gutman et al., 2024).

### 3.3 Measures

All latent constructs were measured with established multi-item scales and supplemented by a behavioral task. Perceived environmental restorativeness was measured at T2 with the 11 items of the Perceived Restorativeness Scale (PRS); state mindfulness was measured at T2 with 10 items from the relevant subscale of the State Mindfulness Scale (SMS); subjective attention restoration was measured at both T2 and T3 with the 8 items of the DSSQ attention subscale; life satisfaction was measured at T3 with the 5 items of the Satisfaction With Life Scale (SWLS); and positive affect was measured at T3 with the 5 items of the PANAS positive affect subscale. Objective attention was assessed by a backward digit span task administered at T1, T2 and T3, and the standing of this task within the design requires statement at the outset. The task was included as a secondary, performance-based indicator rather than as a further test of the mediation chain, a position adopted in advance of analysis upon the ground set out in Section 2.1, namely that perceived and performance-based restoration are known to dissociate and that a mechanism defined over appraisal and awareness therefore carries no commitment to predicting task performance (Johnson et al., 2022). The task serves accordingly to establish whether any restoration is registered outside self-report, and it is reported in Section 4.5 and interpreted in Section 5.2.2 in that capacity. Negative affect was also measured at T3 with the 5 items of the PANAS negative affect subscale, used to test the discriminant validity of positive affect and common method bias, but it was not included in the structural model as an outcome variable of this study; its modeling as a complete well-being dimension is left for future research (see Section 5.5). Control variables included T1 smartphone addiction (SAS-SV), burnout, baseline attention, and baseline well-being, as well as demographic information. Digital disconnection intensity was synthesized into a continuous index from information including T1 screen time, camp compliance records, detox duration, and self-reported difficulty of disconnection, and in the actual analysis it was operationalized continuously as the proportional reduction in screen use from T1 to T2.

The number of items and the internal consistency reliability of each scale are shown in Table 4. The reliabilities of the core constructs were at acceptable levels, with good reliability for perceived restorativeness (*α* = 0.89) and state mindfulness (*α* = 0.86). It should be noted candidly that the reliabilities of T1 baseline attention (*α* = 0.64) and baseline well-being (*α* = 0.69) were below the conventional threshold of 0.70; as these are baseline control variables rather than core outcome variables, their influence is relatively limited, but caution should still be exercised when interpreting the related results.

**Table 4:** Measures and internal consistency reliability.

Construct/variable	Scale	No. of items	Wave	Cronbach’s *α*
Smartphone addiction	SAS-SV	10	T1	0.82
Burnout	Burnout scale	6	T1	0.77
Baseline attention	Baseline attention scale	4	T1	0.64
Baseline well-being	Baseline well-being scale	5	T1	0.69
Perceived environmental restorativeness	PRS	11	T2	0.89
State mindfulness	SMS	10	T2	0.86
Subjective attention restoration	DSSQ-Attention	8	T2	0.81
Subjective attention restoration	DSSQ-Attention	8	T3	0.82
Life satisfaction	SWLS	5	T3	0.77
Positive affect	PANAS-Positive	5	T3	0.80
Negative affect	PANAS-Negative	5	T3	0.81

The sources and versions of the scales were determined according to the study protocol.

The applicability of the perceived restorativeness scale in this study can be referenced against the psychometric validation of the scale completed in recent research (Menardo et al., 2024); its operationalization of the “perceived restorative environment” in tourism settings is also consistent with the measurement practices for this construct in recent tourism research (Jeong, 2024).

Published Chinese versions or Chinese revisions of the instruments were adopted wherever such versions existed, and their item wording was retained without alteration. The DSSQ attention subscale, for which no such version was available, was prepared through forward translation by bilingual researchers, blind back-translation against the source, and resolution of the discrepancies in discussion before administration. The item pool was thereafter reviewed by three experts, whose comments led to the rewording of four items so that the referent was the trip rather than an everyday setting, no item being deleted, no meaning altered and no response format changed. A pilot administration involving 30 travelers drawn from the same population and not retained within the main sample preceded the survey proper, established a mean completion time of approximately 12 min, and led to the simplification of the instructions and to the addition of a prompt marking two reverse-scored items.

Life satisfaction and positive affect were treated as two distinct components of subjective well-being, and this study measured and modeled them as separate constructs; the empirical basis for this treatment is given in the measurement model in Section 4.3.

### 3.4 Data analysis strategy

The data analysis proceeded in predefined steps, designed to address, in sequence, self-selection bias, construct validity, cross-context comparability, mediation mechanisms, and method bias.

Propensity score weighting was used to balance baseline differences between the two groups on observable covariates, and balance was assessed by the standardized mean difference before and after weighting (Chesnaye et al., 2022). After establishing between-group comparability, the fit of the measurement model and the convergent and discriminant validity of the constructs were examined through confirmatory factor analysis. Given that this study used a longitudinal, two-group design, measurement invariance was further tested: configural, loading, and intercept equivalence were examined across groups for all six constructs and across waves for subjective attention, the only construct administered with identical items at both T2 and T3 to ensure that the compared constructs carried comparable meaning at different time points and in different groups; the invariance tests were specified and assessed within the covariance-based structural equation framework, with equivalence judged by the change in comparative fit across nested constraints.

The analysis proceeded along two estimation strategies whose respective roles require statement, since each addresses a threat that the other does not. The mechanism was estimated by latent variable structural equation modeling under robust maximum likelihood with full information maximum likelihood for missing data, which carries the measurement error of the multi-item constructs into the structural estimates and retains at every wave the information contributed by participants lost at a later one, and which models perceived environmental restorativeness and state mindfulness as serial mediators, treating subjective attention, life satisfaction and positive affect as separate outcomes and controlling for the corresponding baseline variable in each case. These models are not weighted. Self-selection was addressed instead through inverse probability of treatment weighting applied to a path analysis conducted upon scale composites and estimated with heteroskedasticity-consistent (HC3) standard errors, which reproduces the same chain under adjustment for the observed covariates, so that the latent models carry the measurement argument while the weighted composite models carry the confounding argument and the convergence of the two is what supports the inference rather than either taken alone. The extent to which this division leaves the mechanism exposed to residual self-selection is stated in Section 5.4. On this basis, the group variable was replaced with continuous digital disconnection intensity to examine the dose–response relationship. The confidence intervals for the indirect effects of serial mediation were estimated with the bias-corrected bootstrap. The “mindfulness-dominant” claim implied by Proposition 3 was tested by comparing the mindfulness-mediated indirect effect with the direct effect of perceived restorativeness bypassing mindfulness.

The treatment of common method bias combined procedural control with statistical testing. The procedural remedies were specified in line with recent methodological reviews of such remedies (Yao and Xu, 2024) and comprised the separation of the mediators at T2 from the outcomes at T3 by an interval of 4 weeks, which removes predictor and criterion from a single retrieval context; the addition of a behavioral task that shares no method with the self-reports; anonymity of response, in that answers were linked across waves by a code rather than by identity and were withheld from camp operators and from the recruiting communities alike, which removes the incentive to answer in a socially desirable direction; the presentation of each scale upon a separate page under its own instruction; and an explicit statement to participants that no answer was right or wrong and that the study concerned their own experience rather than any evaluation of the operator. Statistically, the unmeasured latent method construct (ULMC) technique was used: an orthogonal method factor loaded by all observed indicators was introduced into the measurement model, and whether method variance posed a substantial threat was judged by comparing model fit (Ding et al., 2023). The unmeasured latent method construct was preferred to a single-factor diagnostic on the ground that it models method variance rather than merely inspecting the share of variance carried by an unrotated first factor, a preference consistent with recent systematic reviews of method bias mechanisms and of their procedural and statistical remedies (Yao and Xu, 2024), though the complete-case requirement that the specification imposes limits its reach in the present data (Section 4.3).

Robustness checks included re-estimating the main paths after applying quantile truncation to the IPTW weights, and reverifying the main conclusions with propensity score matching; the robustness rationale for weight truncation and propensity score post-calibration is given in the methodological basis cited above (Gutman et al., 2024).

## 4 Results

### 4.1 Sample characteristics and attrition analysis

Of the 500 participants at baseline, 250 completed all three waves of measurement. To test whether follow-up attrition occurred systematically with baseline characteristics, this study compared the means of T3 completers and non-completers on all T1 variables. The results showed no significant differences between the two on age, screen time, smartphone addiction, burnout, baseline attention, baseline well-being, or backward digit span (all *p* > 0.12), suggesting that follow-up attrition was not systematically associated with observable baseline characteristics and supporting the assumption that the data were approximately missing at random. The attrition comparison for each variable is shown in Table 5.

**Table 5:** Baseline difference tests between T3 completers and non-completers.

T1 variable	Completers *M*	Non-completers *M*	Difference	*p*
Age	34.39	33.78	0.61	0.266
Screen time	8.37	8.33	0.04	0.799
Smartphone addiction (SAS-SV)	3.69	3.61	0.08	0.220
Burnout	3.90	3.82	0.09	0.122
Baseline attention	3.04	3.10	−0.06	0.341
Baseline well-being	3.04	3.02	0.02	0.739
Backward digit span	6.55	6.65	−0.10	0.344

Table 5 shows the completing and the non-completing halves of the baseline sample to be separated by margins that are small in absolute terms as well as statistically indistinguishable, the largest difference amounting to 0.61 years of age, and retention was identical across the two conditions at both follow-ups (Table 2), so that attrition does not stand as a differential process capable of generating the contrasts reported below. The magnitude of the loss nonetheless requires a guarded reading, since baseline equivalence upon measured variables is evidence against selective loss without amounting to a demonstration of its absence, weighting adjusts composition with respect to observed covariates alone (Metten et al., 2022), and a retention rate of one half leaves the outcome models resting upon 250 participants with materially less precision than the enrolled sample would have afforded, a point carried into Section 5.4.

### 4.2 Covariate balance

After inverse probability of treatment weighting, the standardized mean differences (SMDs) of all covariates between the two groups fell below 0.10, meeting the balance criterion. Several covariates with larger pre-weighting differences were effectively corrected after weighting; for example, planned trip days fell from 0.98 to 0.04, T1 screen time from 0.40 to 0.08, and burnout from 0.27 to 0.03. The propensity score matching used for comparison also achieved balance on most covariates, but residual imbalance with SMDs above 0.10 remained on education, marital status, some income and occupation categories, city tier, and baseline attention and well-being. In view of this, this study used IPTW as the primary analysis and matching only as a robustness reference. The balance of representative covariates is shown in Table 1.

### 4.3 Measurement model and construct validity

Confirmatory factor analysis showed that the measurement model fit well, *χ*^2^(887) = 948.15, *χ*^2^/df = 1.07, CFI = 0.988, TLI = 0.987, RMSEA = 0.012 [90% CI (0.000, 0.019)], SRMR = 0.045. The composite reliability (CR) of each construct ranged between 0.76 and 0.89, all at acceptable levels. The average variance extracted of all six constructs fell below the conventional threshold of 0.50, ranging between 0.37 and 0.46, and given that all composite reliabilities exceeded 0.60 convergent validity may be regarded as acceptable under the Fornell–Larcker criterion, though the low values are acknowledged as a measurement limitation of this study. The reliability and validity indices of the constructs are shown in Table 6.

**Table 6:** Construct reliability and convergent validity.

Construct	No. of items	CR	AVE
Perceived environmental restorativeness (PRS)	11	0.89	0.42
State mindfulness (SMS)	10	0.87	0.40
Subjective attention restoration (DSSQ T3)	8	0.82	0.37
Life satisfaction (SWLS)	5	0.76	0.40
Positive affect (PANAS-Pos)	5	0.81	0.46
Negative affect (PANAS-Neg)	5	0.81	0.46

For discriminant validity, the HTMT values between all pairs of constructs were below the threshold of 0.85, the highest being between PRS and SMS (HTMT = 0.72), supporting discriminant validity. It should also be noted that, when examined with the Fornell–Larcker criterion, the latent correlation between PRS and SMS (*r* = 0.71) was slightly higher than the square roots of their AVEs (0.65 and 0.63, respectively), meaning that this pair of constructs did not pass the Fornell–Larcker criterion; all other construct pairs satisfied it. Overall, relying mainly on the more recent and robust HTMT criterion, discriminant validity can be supported, but the high association between PRS and SMS should be kept in mind during interpretation.

A measurement model combining life satisfaction and positive affect into a single well-being factor fits appreciably less well (CFI = 0.914, SRMR = 0.089) than the separate specifications from which the structural models of Section 4.4 are built, each of which fits closely (CFI = 1.000, SRMR = 0.036), so the two outcomes are modelled and reported separately throughout rather than combined into one.

Common method bias was examined with the unmeasured latent method construct technique. Because the specification requires every one of the 44 indicators to be observed for a case to contribute, the test rests upon the 130 participants complete across all indicators rather than upon the full sample carried by the measurement model, and its baseline is correspondingly a different model fitted to a different set of cases, so the comparison is to be read as a sensitivity check upon complete cases rather than as a property of the model reported above. Within that restricted sample, the introduction of an orthogonal method factor loaded by all indicators improved fit only marginally relative to its own baseline (ΔCFI = 0.005, ΔRMSEA = −0.001), which indicates that method variance does not pose a substantial threat to the relationships among the constructs, though the reduced sample limits the weight the diagnostic can bear.

Because this study compares two conditions and, for one construct, two waves, the constructs entering those comparisons were examined for measurement equivalence before their relationships were interpreted. Equivalence was assessed across nested constraints upon configural, loading and intercept structure, with the change in comparative fit taken as the criterion and the change in the root mean square error of approximation reported alongside it. Only subjective attention was administered with identical items at more than one wave, so longitudinal equivalence was testable for that construct alone and was neither examined nor claimed for perceived restorativeness, state mindfulness, life satisfaction or positive affect. The full results are reported in Table A1 of the Appendix and are summarized here.

Table A1 shows the pattern to be selective. The two constructs carrying the mediating chain hold at every level across the conditions, as do subjective attention and positive affect, whereas life satisfaction holds at the loading level and fails at the intercept level (ΔCFI = −0.015, ΔRMSEA = 0.042), and negative affect, which enters no structural model, holds on the comparative fit index while its change in the root mean square error of approximation reaches the boundary (ΔCFI = 0.003, ΔRMSEA = −0.015). Subjective attention holds at the loading level across waves (ΔCFI = −0.003) and fails at the intercept level by a wide margin (ΔCFI = −0.072, ΔRMSEA = 0.030). Because the inferences of this study rest upon structural relationships estimated with the baseline value of each outcome controlled rather than upon comparisons of latent means, the two failures constrain what may be said about levels without bearing upon the paths through which the exposure reaches either.

### 4.4 Main results of the structural model

The three outcomes were each estimated with a latent variable structural equation model under robust maximum likelihood and full information maximum likelihood, without weighting and controlling for the corresponding baseline variable, and the fit was excellent throughout, with CFI = 0.998 and RMSEA = 0.005 for subjective attention, CFI = 1.000 and RMSEA = 0.000 for life satisfaction, and CFI = 1.000 and RMSEA = 0.000 for positive affect.

The path from exposure to perceived restorativeness was highly consistent and strong across the three models (standardized coefficient ≈ 0.52, *p* < 0.001), supporting H1. The path from perceived restorativeness to state mindfulness was equally robust (standardized coefficient ≈ 0.67, *p* < 0.001), supporting H2. The paths from state mindfulness to the three outcomes were consistent in direction and all significant: to subjective attention (standardized 0.36, *p* < 0.001), to life satisfaction (standardized 0.26, *p* = 0.012), and to positive affect (standardized 0.81, *p* < 0.001), supporting H3 and H4.

**Figure 3:**
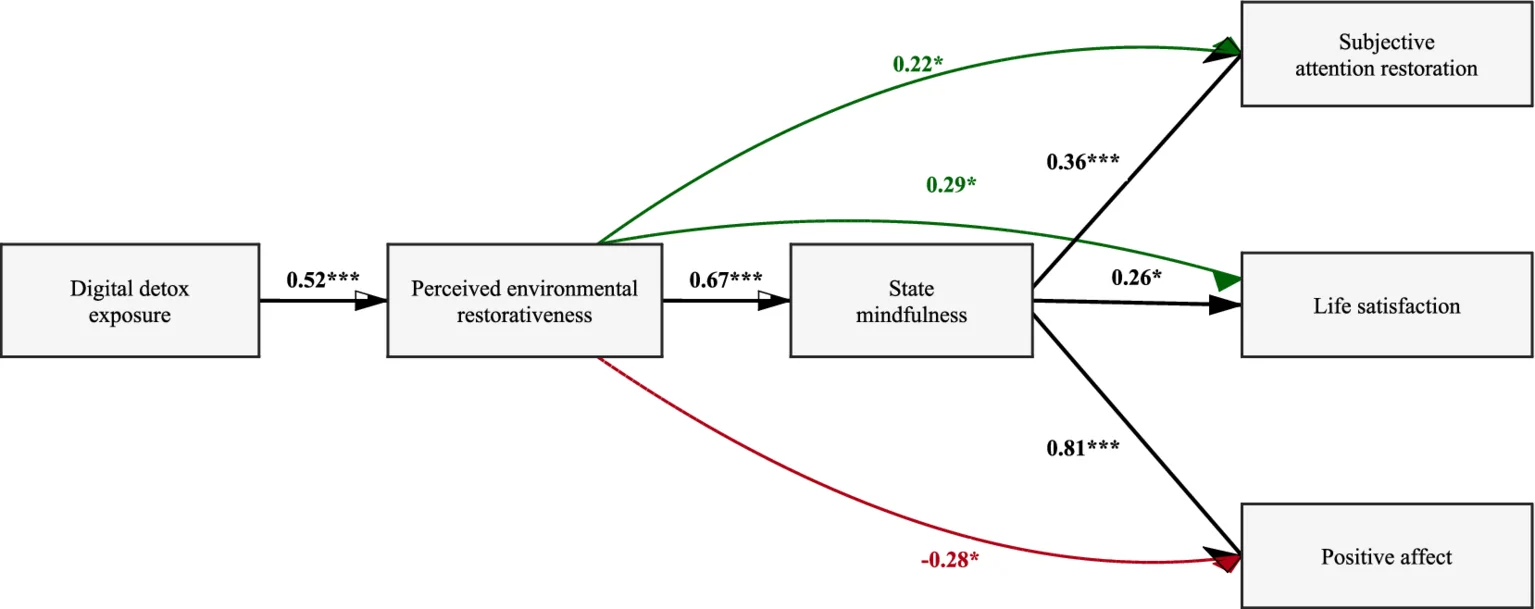
Path diagram of the latent variable structural model (standardized coefficients).

The model also revealed a differentiated pattern in the direct paths from perceived restorativeness to the outcomes. For subjective attention, perceived restorativeness retained a significant direct path beyond mindfulness (standardized 0.22, *p* = 0.025); for life satisfaction, its direct path was positive and significant (standardized 0.29, *p* = 0.014); whereas for positive affect, its direct path was negative and significant (standardized −0.28, *p* = 0.033). In all three models, the direct path from group to outcome was nonsignificant. The key paths of each outcome model are shown in Figure 3, Table 7.

**Table 7:** Key paths of the latent variable structural models (standardized coefficients).

Path	Subjective attention	Life satisfaction	Positive affect
Exposure → perceived restorativeness	0.52^***^	0.52^***^	0.52^***^
Perceived restorativeness → state mindfulness	0.67^***^	0.67^***^	0.67^***^
State mindfulness → outcome	0.36^***^	0.26^*^	0.81^***^
Perceived restorativeness → outcome (direct)	0.22^*^	0.29^*^	−0.28^*^
Group → outcome (direct)	0.05 n.s.	0.03 n.s.	−0.04 n.s.

^*^p < 0.05, ^**^p < 0.01, ^**^p < 0.001; n.s., nonsignificant. Coefficients are taken from the latent variable SEM corresponding to each outcome.

### 4.5 Mechanistic differences between cognitive and affective well-being

Placing the two types of well-being outcomes side by side reveals a clear mechanistic asymmetry. For life satisfaction, perceived restorativeness acts both indirectly through mindfulness and via a positive direct path, showing consistent partial mediation; for positive affect, it is transmitted almost entirely through mindfulness—the effect of mindfulness is very strong (standardized 0.81), whereas the direct path of perceived restorativeness, net of mindfulness, turns negative. The latter constitutes a suppression effect (inconsistent mediation): because perceived restorativeness exerts a strong positive indirect effect on positive affect through mindfulness (approximately 0.67 × 0.81 on the standardized path), its total effect remains positive, but the independent effect on immediate affect of the portion of restorativeness not channeled through mindfulness is negative. This asymmetry indicates that a considered evaluation of life can be enhanced through multiple pathways, whereas present-moment affective pleasure relies more on the single channel of mindfulness; this path pattern is shown in Figure 3.

Objective attention, measured by backward digit span at T3, showed a significant unadjusted difference between the conditions (Cohen’s *d* = 0.32, *p* = 0.014, higher in the detox condition). In the weighted path model, which controls for T1 backward digit span together with the covariates of Table 3, none of the three paths bearing upon it reached significance: the direct path from group (*b* = 0.175, SE = 0.177, *p* = 0.323), the path from perceived restorativeness (*b* = 0.015, SE = 0.129, *p* = 0.907) and the path from state mindfulness (*b* = 0.088, SE = 0.124, *p* = 0.477), and the serial indirect effect was likewise nonsignificant [0.02, 95% CI (−0.07, 0.12); Table 8], so that the restorativeness–mindfulness mechanism posited here does not account for the objective difference. The result is reported in the secondary capacity assigned to the task at the design stage (Section 3.3) and is retained within the interpretation rather than set aside, since the divergence between a difference that is present in performance and a mechanism that fails to explain it bears directly upon the scope of the proposed pathway and is taken up in Section 5.2.2.

**Table 8:** Indirect effects of serial mediation (bias-corrected bootstrap, 5,000 resamples).

Predictor	Outcome	Indirect effect	95% CI	Significant
Exposure (group)	Subjective attention	0.34	[0.14, 0.54]	Yes
Exposure (group)	Life satisfaction	0.24	[0.07, 0.46]	Yes
Exposure (group)	Positive affect	0.75	[0.50, 1.17]	Yes
Exposure (group)	Objective attention (secondary indicator)	0.02	[−0.07, 0.12]	No
Disconnection intensity	Subjective attention	0.07	[0.03, 0.12]	Yes
Disconnection intensity	Life satisfaction	0.051	[0.003, 0.109]	Yes
Disconnection intensity	Positive affect	0.154	[0.094, 0.226]	Yes

The first three rows are unstandardized indirect effects from the latent variable SEMs of Section 4.4, estimated under robust maximum likelihood with full information maximum likelihood and without weighting. The remaining four rows are taken from the composite path analysis, the objective attention row because backward digit span is a single indicator and the three disconnection intensity rows because that analysis carries the continuous operationalization of the exposure. The two sets stand in different metrics and are not to be compared with one another, nor with the standardized coefficients of Table 7. All intervals are bias-corrected bootstrap intervals from 5,000 resamples. Objective attention was a secondary, performance-based indicator (Section 3.3).

### 4.6 Dose–response analysis

The dose–response relationship was examined by a path analysis upon scale composites in which the group variable was replaced by continuous digital disconnection intensity, operationalized as the proportional reduction in screen use from T1 to T2. Disconnection intensity predicted perceived restorativeness (*b* = 0.46, *p* < 0.001) and perceived restorativeness in turn predicted mindfulness (*b* = 0.61, *p* < 0.001), while the direct paths from disconnection intensity to subjective attention (*b* = 0.002, *p* = 0.976), to life satisfaction and to positive affect were all nonsignificant once the mediators were included, whereas the serial indirect effect reached each of the three, which indicates that the association between disconnection intensity and the outcomes runs through the restorativeness–mindfulness chain rather than around it.

The bearing of this pattern upon the exposure requires one further test, since disconnection intensity varies across the two conditions as well as within them and an association estimated over the sample as a whole cannot distinguish the degree of disconnection from the conditions under which it was attained. The analysis was accordingly repeated within the detox condition alone, where the camp setting holds the environmental and social conditions approximately constant while the degree of disconnection continues to vary. Disconnection intensity did not predict perceived restorativeness within that condition, whether estimated without covariates (*b* = 0.23, SE = 0.36, *p* = 0.515, *R*^2^ = 0.003) or with T1 screen time, smartphone addiction, burnout, baseline attention and baseline well-being entered as controls (*b* = 0.22, SE = 0.49, *p* = 0.648, *R*^2^ = 0.018). The restricted range of the index within the detox condition and the reduced sample of 150 both lower the power of this test, so the result is not to be read as evidence of no relationship, yet the point estimate stands close to zero relative to its standard error and the outcome cannot be attributed to power alone. What the dose evidence reported above establishes is therefore a gradient carried by variation between the conditions rather than within them, which bounds what the continuous operationalization can show about digital disconnection considered apart from the setting in which it occurs, and Section 5.4 states the consequence.

The pattern remains consistent with the group model in the location it assigns to the effect, namely within the mediating chain rather than around it, and it compensates in part for the identification limitation introduced by the absence of a no-travel baseline without compensating for the conjunction of disconnection with the conditions that accompany it.

### 4.7 Tests of mediation effects

The indirect effects of serial mediation were estimated with the bias-corrected bootstrap (5,000 resamples). The results showed that the indirect effects through the “exposure → perceived restorativeness → mindfulness” chain were significant for subjective attention [indirect effect 0.34, 95% CI (0.14, 0.54)], life satisfaction [0.24, 95% CI (0.07, 0.46)], and positive affect [0.75, 95% CI (0.50, 1.17)], supporting H5 for these three outcomes. With continuous disconnection intensity as the predictor, the indirect effects on subjective attention [0.07, 95% CI (0.03, 0.12)], on life satisfaction [0.051, 95% CI (0.003, 0.109)] and on positive affect [0.154, 95% CI (0.094, 0.226)] were likewise significant.

The serial indirect effect on objective attention (backward digit span) was nonsignificant [0.02, 95% CI (−0.07, 0.12), interval containing zero], meaning that H5 did not hold for objective attention, consistent with Section 4.5. The indirect effects are shown in Table 8.

### 4.8 Competing paths and robustness

H6 compares two pathways that share perceived environmental restorativeness as the starting point: the indirect effect through “perceived restorativeness → state mindfulness → outcome,” and the direct path “perceived restorativeness → outcome” that bypasses mindfulness (Table 7). For positive affect the mindfulness-mediated indirect effect significantly dominated the negative direct path and H6 received strong support, whereas for subjective attention and life satisfaction perceived restorativeness retained a significant positive direct path beyond mindfulness (Table 7) and the indirect effect did not dominate it, so H6 received only partial support. A further characterization of mediation strength requires distinguishing mediation types. Subjective attention showed consistent mediation (indirect and direct paths in the same direction); the variance accounted for by the restorativeness–mindfulness chain in the composite path analysis stood at 0.22 with group as predictor and at 0.34 with disconnection intensity as predictor, a proportion not to be confused with the latent indirect effects reported in Table 8. Life satisfaction likewise showed consistent partial mediation. Positive affect shows inconsistent mediation of the kind described in Section 4.5, for which the variance accounted for does not hold mathematically, so the indirect effect (Table 8) and the negative direct path are presented separately rather than as a proportion mediated.

Robustness checks indicated that the main conclusions were stable. After applying 1/99 and 5/95 quantile truncation to the IPTW weights and re-estimating the main paths, the direction and significance of the core coefficients were largely unchanged. One change worth recording candidly is that the direct path from group to mindfulness changed from marginally nonsignificant (*p* = 0.070) to significant (*b* = 0.30, *p* = 0.030) under 5/95 truncation, suggesting that this direct path is borderline and sensitive to weight handling, and should not be regarded as robustly significant when interpreted. The results reverified with propensity score matching were consistent in direction with the primary analysis.

## 5 Discussion

### 5.1 Overview of main findings

Using a two-group quasi-experiment combined with a three-wave longitudinal and dose–response design, this study examined the mechanisms through which digital detox tourism affects attention restoration and two types of subjective well-being via perceived environmental restorativeness and state mindfulness. The results place the effect of digital detox exposure within the restorativeness–mindfulness chain rather than around it, since the serial indirect effects were confirmed by bootstrapping for all three outcomes while the direct paths from exposure and from disconnection intensity to the outcomes were not, and from disconnection intensity to the outcomes were not. These results provide empirical support for the core mechanism of the dual-disconnection hypothesis, but they show differences across well-being dimensions that warrant closer interpretation.

### 5.2 Theoretical contributions

#### 5.2.1 Attention restoration theory in a setting of dual digital–physical disconnection

The contribution of this study to attention restoration theory is a contextual one and is best stated in bounded terms. The “being away” dimension was formulated for physical displacement, and what the present evidence adds is that where digital withdrawal accompanies physical withdrawal the setting is appraised as more restorative and the strength of that appraisal rises with the degree of withdrawal across the sample as a whole. The dose evidence does not extend to variation within the detox condition, where disconnection intensity ceased to predict the appraisal once the setting was held approximately constant (Section 4.6), so the reading that digital disconnection functions as a form of psychological distancing in its own right is supported by the group contrast and by the sample-wide gradient without being confirmed at the level where it would be decisive. Several theoretically grounded accounts may explain this null within-camp gradient. A threshold effect is plausible: once the sealed-device protocol has imposed a substantial disconnection, further marginal reductions in residual screen time may no longer yield additional restorative benefit, consistent with evidence that the dose–response curve between nature exposure and restoration flattens at higher doses (Bell et al., 2025). The narrow range of disconnection intensity that remains after the protocol has removed the bulk of screen use further compresses the predictor distribution, reducing the statistical power to detect any residual gradient. Most consequentially, the detox camp is a composite experience in which digital disconnection co-occurs with intensive nature immersion, structured physical activity, and collective social engagement (Section 3.1); once these shared conditions are held constant, screen-time reduction alone may be too small a source of variation to register an independent contribution. This reading aligns with Proposition 2, which frames the detox stay as a package whose digital and environmental elements are conjoined, and it renders the null within-camp finding theoretically informative—demarcating the point at which disconnection ceases to act as an independent incremental driver and instead functions as one component of a broader restorative bundle. What the study establishes is accordingly an extension of the theory’s application to a class of setting rather than a revision of its structure, since no element of the original account is displaced and the mechanism carrying the effect remains the one the theory already specifies. This finding echoes recent meta-analyses that characterize the restorative effect of nature exposure as a dose function—the latter argue that exposure duration, as a “dose,” moderates the strength of the restorative effect (Bell et al., 2025). The present study carries that dose logic from exposure duration across to digital disconnection intensity as a description of the sample-wide gradient, which supplies empirical content for the applicable boundary of restoration theory in the digital age without establishing the gradient within a fixed setting and without licensing extrapolation beyond the range observed here.

#### 5.2.2 Mindfulness as the core pathway from restoration to well-being

This study confirms that state mindfulness is the core mediator through which perceived restorativeness is transformed into well-being. This result is consistent with research that regards mindfulness as the key mechanism transforming restorative experience into psychological benefits (Zeng et al., 2026), and aligns with foundational accounts of mindfulness as a central pathway of psychological change across contexts (Michalak et al., 2011). In the tourism context, the finding further echoes recent evidence that treats present-moment engagement states such as flow as mediators of psychological recovery in tourism (Song et al., 2025), jointly pointing to a process-based explanation of “restorative environment—present-moment awareness—psychological benefit,” rather than treating the effect of the environment on well-being as direct and unmediated.

The scope of this pathway is bounded in a manner that the objective attention result makes visible. A between-condition difference in backward digit span was present, yet neither restorativeness nor mindfulness accounted for it, and the more informative reading is not that the difference should be discounted but that the restorativeness–mindfulness chain is a mechanism of experienced restoration rather than of attentional capacity as a task elicits it. The two need not coincide, since an environment appraised as restorative and attended to mindfully alters the manner in which attentional effort is subjectively borne without guaranteeing a change in the span of what can be held and reversed under instruction, and the dissociation reported in controlled settings anticipates precisely this (Johnson et al., 2022). A performance difference is therefore likely to be carried by routes running alongside the appraisal–awareness sequence rather than through it, none of which was measured here, so the divergence argues for the addition of pathways to the model rather than for the sufficiency of the one tested, and it qualifies the claim that mindfulness is the core mediator, which holds for the outcomes as experienced and reported and remains untested for attention as performance defines it.

#### 5.2.3 Mechanistic asymmetry between cognitive and affective well-being

The most novel finding of this study is the mechanistic asymmetry between life satisfaction and positive affect. This differentiation emerges only after subjective well-being is decomposed into cognitive and affective components, confirming the argument that the two, though related, differ in nature and antecedents (Fors Connolly and Gärling, 2024). Likewise, recent research that models different dimensions of subjective well-being separately also finds that cognitive and affective components are often driven by different antecedents (King et al., 2024), and the effects of mindfulness interventions on the various dimensions of well-being are not uniform (Scafuto et al., 2025). On this basis the restorativeness–mindfulness mechanism does not act homogeneously across well-being dimensions, in the manner set out in Section 4.5, which is a refined test of Proposition 3 rather than a blanket confirmation of its “mainly through mindfulness” claim.

### 5.3 Practical implications

The findings of this study provide actionable guidance for the design of digital detox tourism products. Given that the effect is transmitted mainly through perceived restorativeness and mindfulness, camp design would be better organized around the conditions that feed those two states than around physical departure alone, which directs attention towards the intensity of natural elements, the thoroughness of digital shielding and the provision of mindfulness guidance. None of the three was experimentally manipulated here, the first two are in any case conjoined in this design and cannot be credited separately, and the three are accordingly advanced as design hypotheses that follow from the estimated mechanism and await a study that varies them deliberately. The dose–response result describes a gradient across the sample as a whole and does not reappear within the detox condition, so it distinguishes a thorough disconnection from a partial one without establishing that further tightening of the protocol within a camp would yield further restorative gain, and no recommendation upon the degree of shielding required can rest upon it. At the same time, the mechanistic difference between cognitive and affective well-being suggests to practitioners that, if the goal is to enhance travelers’ present-moment affective experience, the mindfulness guidance component is especially critical, whereas if the goal is to improve their overall evaluation of life, the restorative environment itself can also operate through non-mindfulness pathways. This product design approach, grounded in the relationship between restorative environments and tourists’ psychological benefits, is consistent with the recent orientation in tourism research that incorporates perceived restorative qualities into the mechanisms of destination experience and loyalty (Zhu et al., 2025). At the policy level, the conclusions of this study can connect with the agendas on mental health and stress relief in UN Sustainable Development Goal 3 and Healthy China 2030, which suggests digital detox tourism as an avenue of psychological recovery worth policy attention, although the present sample is self-selected and occupationally narrow and the connection is therefore offered as a direction for further examination rather than as an estimate of benefit at the level of a population.

### 5.4 Limitations

This study has several limitations that must be acknowledged explicitly. The absence of a no-travel control means that the contribution of a general vacation effect cannot be ruled out entirely, and although the dose–response analysis supplies compensatory evidence it does not stand in for a true no-travel baseline. A related boundary is set by the comparison actually drawn, since screen use in the comparison condition also fell substantially across the trip (Table 2), so that what the group contrast estimates is the increment of a thorough disconnection over the partial disconnection that ordinary leisure travel already produces rather than the effect of disconnection measured against continued connectivity.

The comparison holds the fact of travel constant and does not hold the intensity of contact with nature constant, since digital withdrawal, immersion in nature, physical activity, collective work and the form of accommodation all vary together across the conditions (Section 3.1). The dose–response analysis was intended to bear upon this conjunction and does not resolve it, since disconnection intensity ceased to predict perceived restorativeness once the analysis was confined to the detox condition, where the setting is approximately constant (Section 4.6), so the association between the degree of disconnection and the restorative appraisal is carried by variation between the conditions rather than within them. Restricted range and a sample of 150 lower the power of that test, which is therefore not evidence of no relationship, yet neither is it the within-condition confirmation the design would have required. What this study identifies is accordingly the effect of a detox stay taken as a composite, and the contribution of digital disconnection considered apart from the conditions delivering it remains open.

Assignment followed from the traveler’s own booking decision, and weighting balances the covariates that were measured without addressing confounding by characteristics that were not, a threat whose direction is foreseeable in that travelers choosing a detox stay are likely to differ in affinity for nature, in motivation to disconnect and in habits of living that bear upon restoration, any of which would move the mediators in the same direction as the exposure. Prior detox experience, smartphone addiction and burnout stand proxy for part of this disposition, yet the disposition itself was not measured, so the estimates are to be read as the effect of the exposure among travelers already disposed to seek it. The division of labour between the two estimation strategies leaves a further gap, in that the latent models are unweighted while the weighted models rest upon composites, so the case rests upon their convergence rather than upon either alone. The three-day floor is an operational criterion, so stays shorter than 3 days are absent from the observed dose range; retention to the final wave was one half of the enrolled sample, and although completers and non-completers proved indistinguishable upon every measured baseline variable (Section 4.1), attrition of this magnitude remains a threat that baseline equivalence upon observed variables cannot discharge; and participants in the detox condition were nested within three camps whose delivery the design does not separate from the protocol, no variance being partitioned at the camp level.

At the measurement level, the average variance extracted of all six constructs fell below the conventional threshold, the latent correlation between perceived restorativeness and state mindfulness (*r* = 0.71) exceeded the square roots of their average variances extracted (0.65 and 0.63), so this pair fails the Fornell–Larcker criterion even as it satisfies the heterotrait–monotrait criterion upon which the discriminant claim principally rests, and the scalar invariance of life satisfaction across conditions and of subjective attention across waves did not hold (Table A1), so latent mean comparisons are to be treated with caution, a limitation mitigated by resting the inferences upon structural relationships instead. The chain carrying the main conclusions is composed almost entirely of self-reports from the same participants, and although the mediators and the outcomes were 4 weeks apart, the method factor test rests upon the 130 participants complete across all indicators (Section 4.3) and carries limited evidential weight on that account, so it is not offered as a demonstration that common method variance has been excluded, such remedies attenuating that variance without eliminating it, and the one indicator not drawn from self-report did not follow the same mechanism. Objective attention showed a significant raw difference between conditions that this mechanism did not explain, and the account offered in Section 5.2.2 identifies unmeasured routes as the likely carriers of that difference without testing them, so the mechanism established here is supported for the outcomes as experienced and reported and untested for attention as performance defines it. The distinction between state and trait mindfulness further suggests that a state measure taken at a four-week follow-up may not reflect more stable trait change (Borgdorf et al., 2025), the negative direct path from perceived restorativeness to positive affect was robustly present within the separate model yet its substantive meaning as a suppression effect calls for further clarification, and the sample was drawn by convenience from a single cultural setting and from a predominantly urban and white-collar population, which bounds the generalizability of the conclusions.

Screen use in the detox condition had returned to 7.12 h daily by the four-week follow-up against 8.59 at baseline (Table 2), so the outcomes measured at T3 were reported by participants who had largely resumed their habitual engagement, which means the effects observed are those that survive a return to ordinary conditions rather than those obtaining while the exposure is in force.

### 5.5 Future research directions

Future research can be extended in the following directions. In terms of design, a randomized trial assigning digital disconnection intensity would identify causality more rigorously, a no-travel baseline would separate the general vacation effect, and a comparison condition matched upon immersion in nature and upon the structure of daily activity would isolate the digital element that the present contrast leaves conjoined with its setting. A design measuring the mediators and the outcomes at every wave would further support a random-intercept cross-lagged panel model, which distinguishes within-person change from stable between-person differences and thereby tests the temporal ordering that the present study assumes, and a specification able to carry sampling weights into a latent model would unite the measurement correction with the adjustment for self-selection that are here divided between two analyses. A multilevel specification would accommodate the clustering of participants within camps and would allow the delivery of a camp to be distinguished from the protocol it delivers, and the exposure itself would be measured more securely by usage records exported from the device than by figures transcribed by the participant. In terms of mechanism, the asymmetry between cognitive and affective well-being deserves reverification over a longer follow-up window to examine its stability, and the in-depth evolution of restorative experience could be further characterized through the process perspective of tourism recovery experience moving from “restoration” to “transformation” (Bai et al., 2025). Mediators that the present model sets aside, among them reduced rumination, psychological detachment and perceived autonomy, could be entered alongside restorativeness and mindfulness so as to establish whether the chain proposed here retains its explanatory role once plausible competitors are admitted to it. In terms of measurement, construct validity evidence should be strengthened and low-AVE scales improved, while more complete well-being dimensions such as negative affect should be incorporated. In terms of external validity, replication studies with cross-cultural and diverse samples will help test the generalizability of the present conclusions.

## 6 Conclusion

Drawing on attention restoration theory and the mindfulness–well-being framework, this study used a two-group quasi-experiment combined with a three-wave longitudinal and dose–response design to examine the mechanisms of digital detox tourism. The results show that digital detox exposure significantly increases perceived environmental restorativeness, and restorativeness, through state mindfulness, in turn promotes subjective attention restoration, life satisfaction, and positive affect; the indirect effects of all three serial mediation chains are confirmed, and the effects rise with digital disconnection intensity across the sample while that gradient does not reappear within the detox condition, so the dual-disconnection hypothesis is supported at the level of the contrast between a thorough and a partial disconnection rather than at the level of disconnection considered apart from its setting. The study further reveals a mechanistic asymmetry between cognitive and affective well-being: life satisfaction is driven by both the direct effect of restorativeness and the indirect transmission through mindfulness, whereas positive affect is realized almost entirely through mindfulness, confirming that the two are related yet mechanistically distinct.

This study carries the “being away” dimension of attention restoration theory into a setting of dual digital–physical disconnection and, through a design that sets a detox stay against concurrent ordinary leisure travel, separates the restorative contribution of such a stay from the vacation effect that travel affords in general, which addresses the problem of insufficient empirical operationalization in prior research within the bounds of one quasi-experiment conducted upon travelers who had chosen such a stay for themselves and under conditions in which disconnection and immersion in nature were delivered together. Constrained by the lack of a “no-travel” baseline, convenience sampling and a single culture, and the failure of the mechanism to explain objective attention, the generalizability of the conclusions remains to be tested. Future research should use controlled trials that randomly assign disconnection intensity, extend the follow-up window, and incorporate cross-cultural samples, in order to confirm the restorative mechanism of digital detox tourism more rigorously.

## Data Availability

The raw data supporting the conclusions of this article will be made available by the authors, without undue reservation.

## Appendix

**Table A1:** Measurement invariance across conditions and across waves.

Panel A. Across conditions (digital detox vs ordinary leisure)						
Construct	Level	CFI	RMSEA	ΔCFI	ΔRMSEA	Supported
Perceived restorativeness	Configural	1.000	0.000	—	—	Yes
	Loading	1.000	0.000	0.000	0.000	Yes
	Intercept	1.000	0.000	0.000	0.000	Yes
State mindfulness	Configural	0.997	0.015	—	—	Yes
	Loading	0.997	0.013	0.000	−0.002	Yes
	Intercept	1.000	0.000	0.003	−0.013	Yes
Subjective attention (T3)	Configural	1.000	0.000	—	—	Yes
	Loading	1.000	0.000	0.000	0.000	Yes
	Intercept	1.000	0.000	0.000	0.000	Yes
Life satisfaction	Configural	0.996	0.027	—	—	Yes
	Loading	1.000	0.000	0.004	−0.027	Yes
	Intercept	0.985	0.042	−0.015	0.042	No
Positive affect	Configural	1.000	0.000	—	—	Yes
	Loading	1.000	0.000	0.000	0.000	Yes
	Intercept	1.000	0.000	0.000	0.000	Yes
Negative affect	Configural	0.997	0.027	—	—	Yes
	Loading	0.996	0.027	−0.001	0.001	Yes
	Intercept	0.999	0.012	0.003	−0.015	Borderline

Panel B. Across waves, subjective attention (T2 and T3)					
Level	CFI	RMSEA	ΔCFI	ΔRMSEA	Supported
Configural	0.998	0.008	—	—	Yes
Loading	0.995	0.011	−0.003	0.003	Yes
Intercept	0.923	0.041	−0.072	0.030	No

Models were estimated under robust maximum likelihood with full information maximum likelihood for missing data and were not weighted (*N* = 500). Delta values are computed against the preceding, less constrained level, and equivalence is judged by the change in comparative fit, with a change of 0.010 or less taken as support. Negative affect enters the measurement model for the assessment of validity and enters no structural model. Longitudinal equivalence was testable only for subjective attention, the sole construct administered with identical items at both T2 and T3.
